# Supplementary material for: Attenuation of phytofungal pathogenicity of Ascomycota by autophagy modulators
Source: Nat Commun. 2024 Feb 29;15:1621. doi: 10.1038/s41467-024-45839-2 (PMC10904834; doi:10.1038/s41467-024-45839-2)
Supplement: Supplementary file 1 — Supplementary Information [file 41467_2024_45839_MOESM1_ESM.pdf]

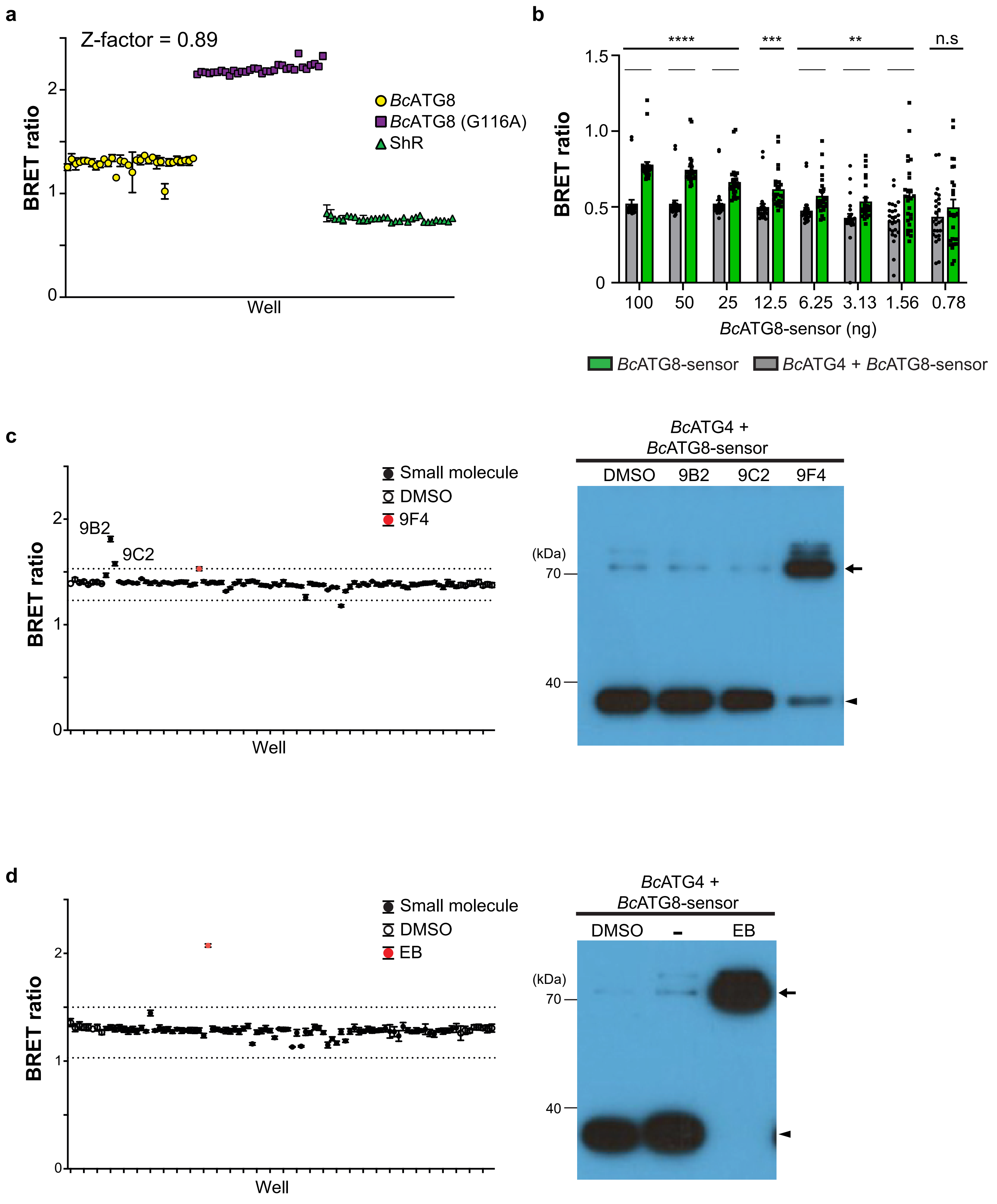

a
Z-factor = 0.89
BRET ratio
Well
BcATG8
BcATG8 (G116A)
ShR
b
BRET ratio
****
***
**
n.s
100
50
25
12.5
6.25
3.13
1.56
0.78
BcATG8-sensor (ng)
BcATG8-sensor
BcATG4 + BcATG8-sensor
c
Small molecule
DMSO
9F4
9B2
9C2
BRET ratio
Well
BcATG4 +
BcATG8-sensor
DMSO
9B2
9C2
9F4
(kDa)
70
40
d
Small molecule
DMSO
EB
BRET ratio
Well
BcATG4 +
BcATG8-sensor
DMSO
-
EB
(kDa)
70
40

e

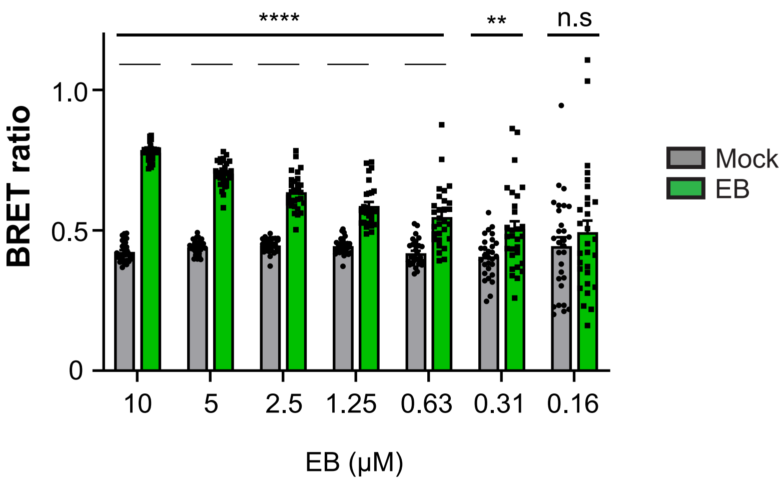

Supplementary Fig. 1. Confirmation of selected hit compounds on the cleavage of *Bc*ATG8 by *Bc*ATG4.

a, High Z-factor suggests excellent quality of HTS with the BRET-based *Bc*ATG8 sensor. The non-cleavable *Bc*ATG8 (G116A) and ShR are used as a positive and a negative control, respectively. n=3 independent experiments. **b,** The detection sensitivity of HTS with the *Bc*ATG8-sensor. The HTS platform with the *Bc*ATG8-sensor is highly sensitive to monitor the maturation of ~1.6 ng of the *Bc*ATG8-sensor (177.3 nM) by ~3.1 ng of *Bc*ATG4. Multiple t-test. Exact p-values are provided in the Source data file. A different number of asterisks indicates statistically significant differences. n=3. **c,** In HTS, the BRET ratio between maximum +0.1 and minimum -0.1 of the DMSO controls is considered not significant (within dotted lines, control zone). Circles and dots represent the DMSO control and the chemical treatment, respectively. Chemicals above the control zone and below the control zone are considered inhibitors and activators, respectively. 9F4 (red dot) is considered as a mild inhibitor according to the BRET ratio. In the same plate, 9B2 and 9C2 show higher BRET ratios. However, only 9F4 is confirmed as an inhibitor by in vitro cleavage assay (right panel). **d,** EB is screened with the highest BRET ratio in the screened plate (left panel, red dot). Confirmation of EB inhibitory effect on the *Bc*ATG4-mediated processing of *Bc*ATG8 by in vitro cleavage assay (right panel). The hyphenated column in the middle of the blot presents one of the first screened candidates from a different plate. Arrow and arrowhead indicate the full-length BRET-sensor and the cleavage byproduct, respectively. **e,** The minimum concentration of EB detected by HTS with the *Bc*ATG8-sensor. The treatment of ~0.3 μM EB causes to increase the BRET ratio that indicates inhibition of the *Bc*ATG4-mediated cleavage of the *Bc*ATG8-sensor, compared to that of mock treatment. Multiple t-test. Exact p-values are in the Source data file. A different number of asterisks indicates statistically significant differences. n=3. n.s indicates not significant. All graphs show means with SE.

a

Inhibitor

Pubchem CID (ΔBRET)	Structure	Pubchem CID (ΔBRET)	Structure	Pubchem CID (ΔBRET)	Structure
3096080 (0.39)	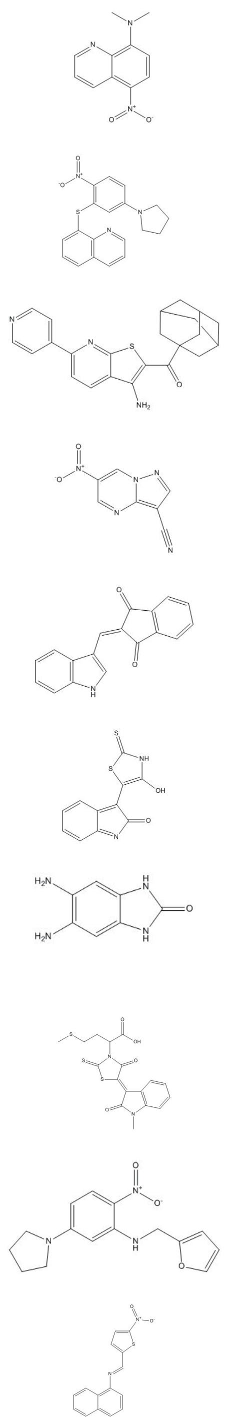	2888142 (0.15)	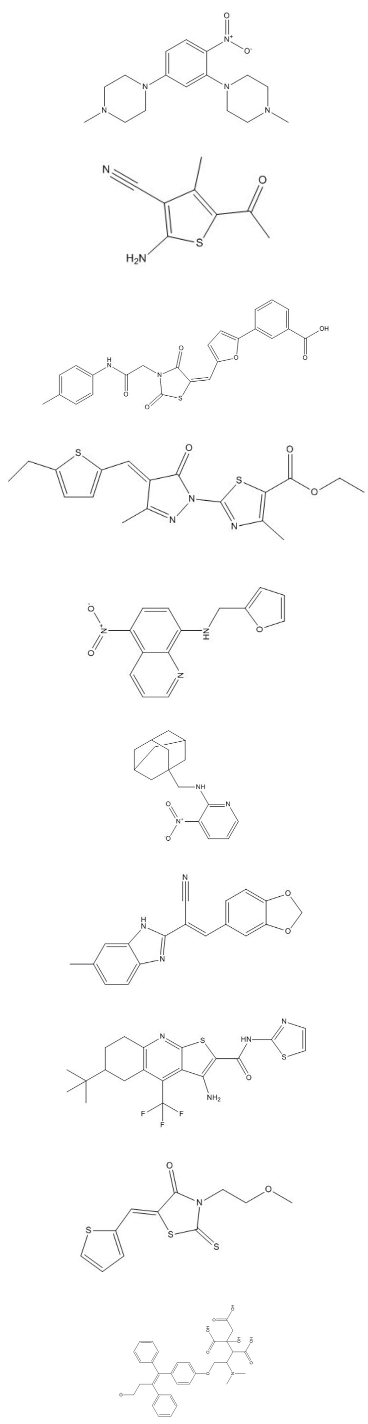	3142724 (0.24)	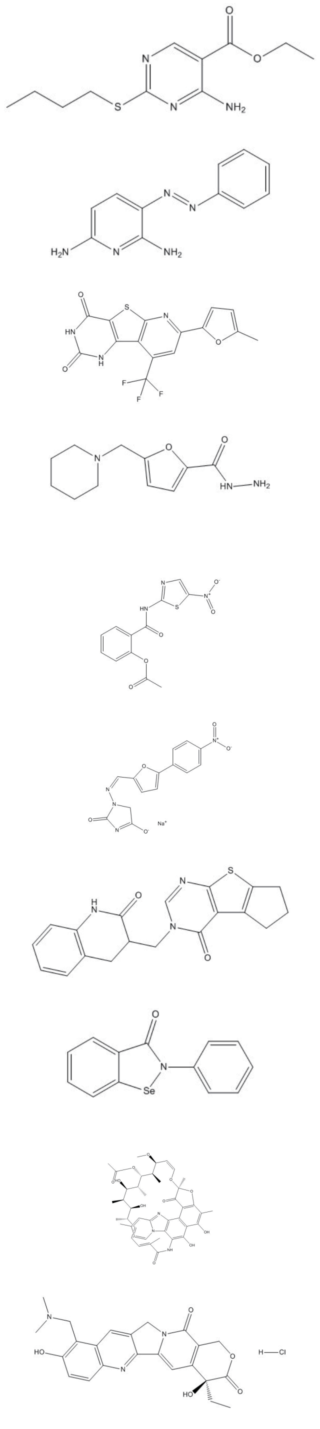
2854998 (0.26)		675983 (9F4) (0.1)		4756 (0.82)	
2961859 (0.64)		1796713 (0.3)		6499580 (0.14)	
670555 (0.27)		5937751 (0.27)		3621158 (0.24)	
280323 (0.28)		2943326 (0.35)		41684 (0.34)	
1269517 (0.25)		5299029 (0.36)		5702224 (0.22)	
727371 (0.11)		5347141 (0.36)		24208275 (0.1)	
3150762 (0.14)		3767434 (0.44)		3194 (EB) (0.71)	
556945 (0.2)		2179877 (0.18)		72111 (0.5)	
314707 (0.38)		3005572 (0.65)		60699 (0.22)	

b

Activator

Pubchem CID (ΔBRET)	Structure	Pubchem CID (ΔBRET)	Structure	Pubchem CID (ΔBRET)	Structure
3218 (-0.19)	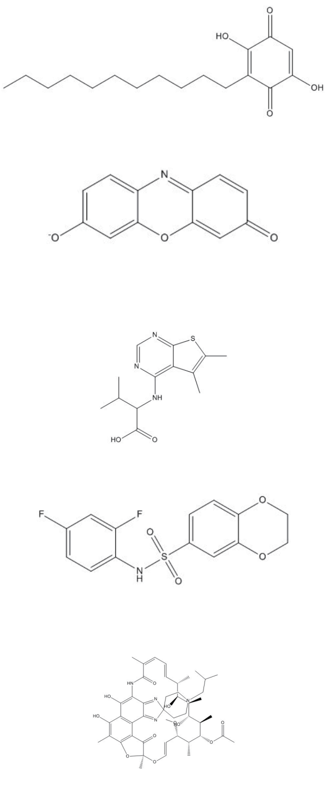	3124342 (-0.26)	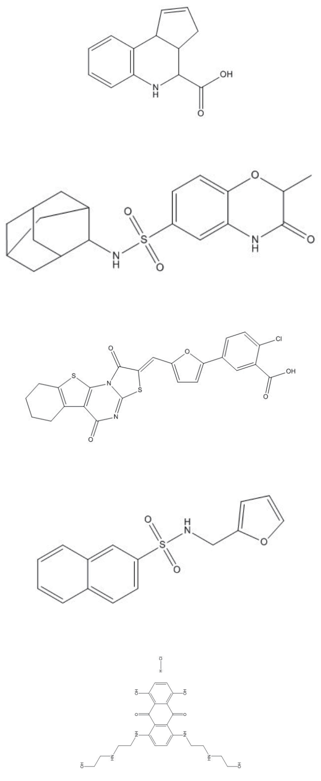	4916039 (-0.16)	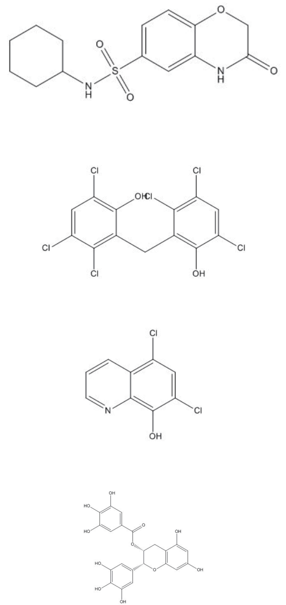
3566705 (-0.17)		24208130 (-0.17)		3598 (-0.21)	
3151025 (-0.22)		5292584 (-0.22)		2722 (-0.27)	
1075754 (-0.2)		683661 (-0.13)		65064 (-0.3)	
145415564 (-0.15)		5458171 (-0.21)			

Supplementary Fig. 2. List of 1st hit compounds screened by the target-based HTS with the *Bc*ATG8 synthetic sensor.

30 inhibitors (**a**) and 14 activators (**b**) are screened by BRET ratio. All inhibitors were subjected to further confirmation by *in vitro* cleavage assay. Two confirmed inhibitors, 9F4 and EB, are marked in red. ΔBRET indicates the difference of the BRET ratio between the chemical treatment and the maximum (or minimum) of the control. Positive (**a**) and negative (**b**) values are considered inhibitors and activators, respectively.

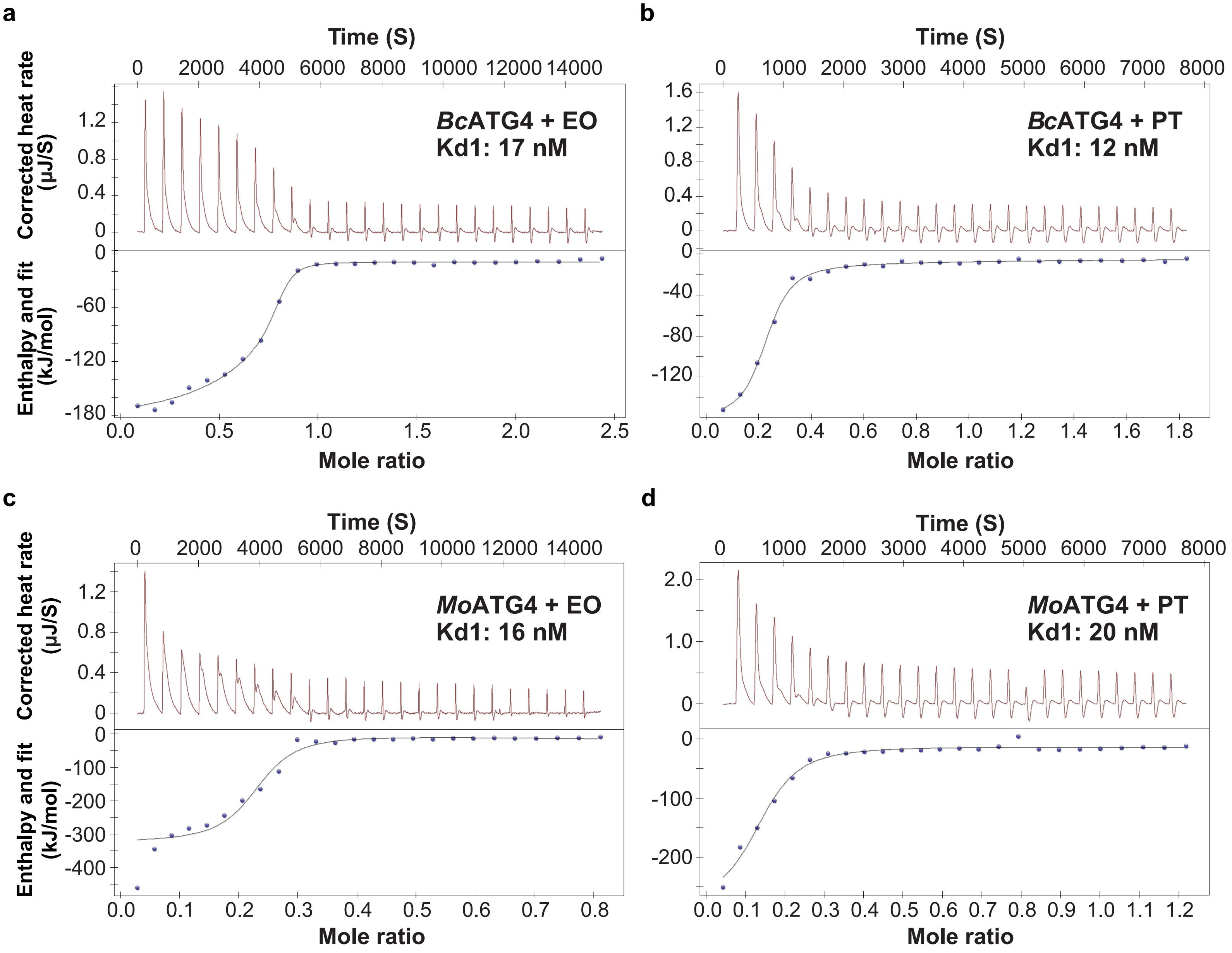

Supplementary Fig. 3. Confirmation of direct binding and affinity of EO and PT to *Bc*ATG4 and *Mo*ATG4 by ITC.

The K_d constants of EO and PT with *Bc*ATG4 and *Mo*ATG4 are estimated by ITC. The ITC data of EO and PT with fungal ATG4s are fitted to the model of multiple binding sites. K_d values of EO with *Bc*ATG4 (**a**) and *Mo*ATG4 (**c**) are 17 and 16 nM and the values of PT are 12 (**b**) and 20 nM (**d**), respectively. All ITC experiments were performed twice. Each representative titration result is shown.

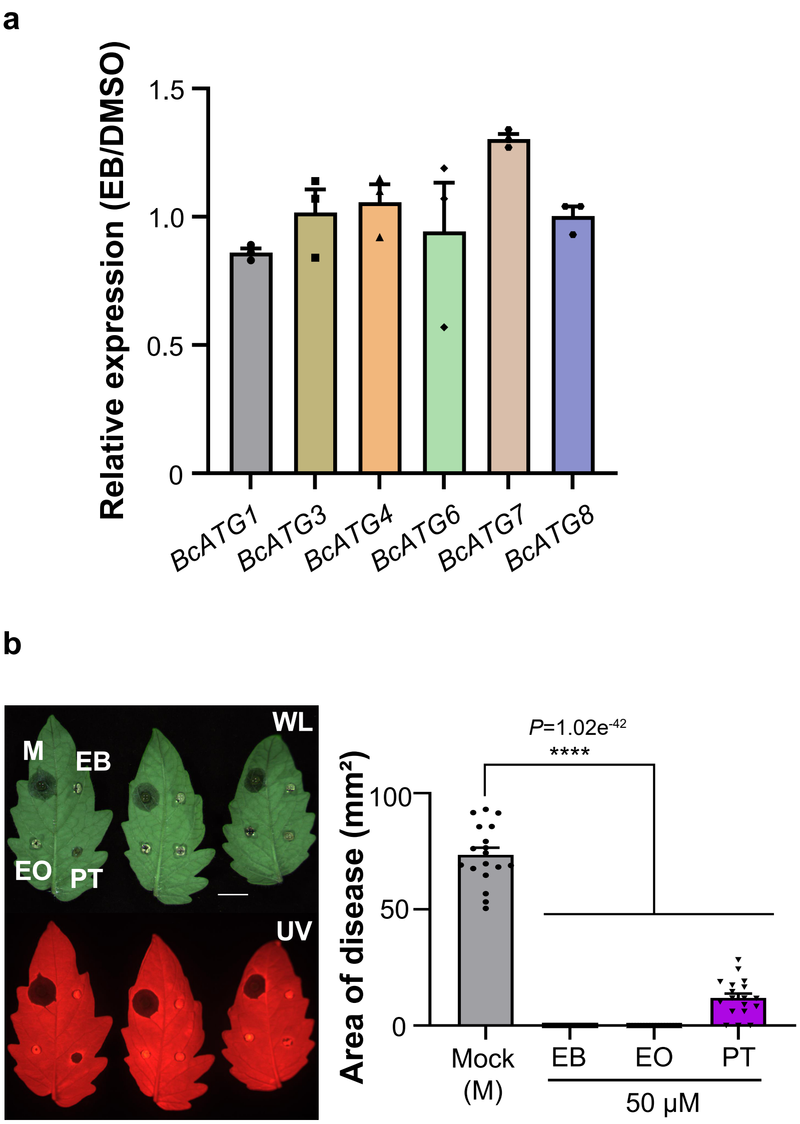

Supplementary Fig. 4. EB effects on transcription of known autophagy genes and pathogenicity of the transgenic *B. cinerea.*

a, 20 μM EB does not alter transcript levels of genes encoding known autophagy core components in *B. cinerea*. Transcript levels of the autophagy genes are estimated by qRT-PCR. The transcription of *BcATG1, BcATG3, BcATG4, BcATG6, BcATG7,* and *BcATG8* is not changed under EB treatment of which condition is the same as explained in the super-resolution microscopy experiment (Fig. 2b). n=3. **b,** Treatment with EB and its analogs inhibits growth of the transgenic *B. cinerea* on tomato leaves. Treatment with 50 μM EB, EO, and PT suppresses the infection by *B. cinerea* harboring the *GFP-BcATG8* cassette compared to the mock control (M) on tomato leaves (left panel). Quantification of disease lesion areas is shown in the right panel. WL, white light; UV, UV light. Scale bar, 1 cm. Error bar, SE. ****, $p < 0.0001$, Dunnett's multiple comparisons test with one-way ANOVA. The three biologically independent experiments were conducted with similar results. A representative image is shown.

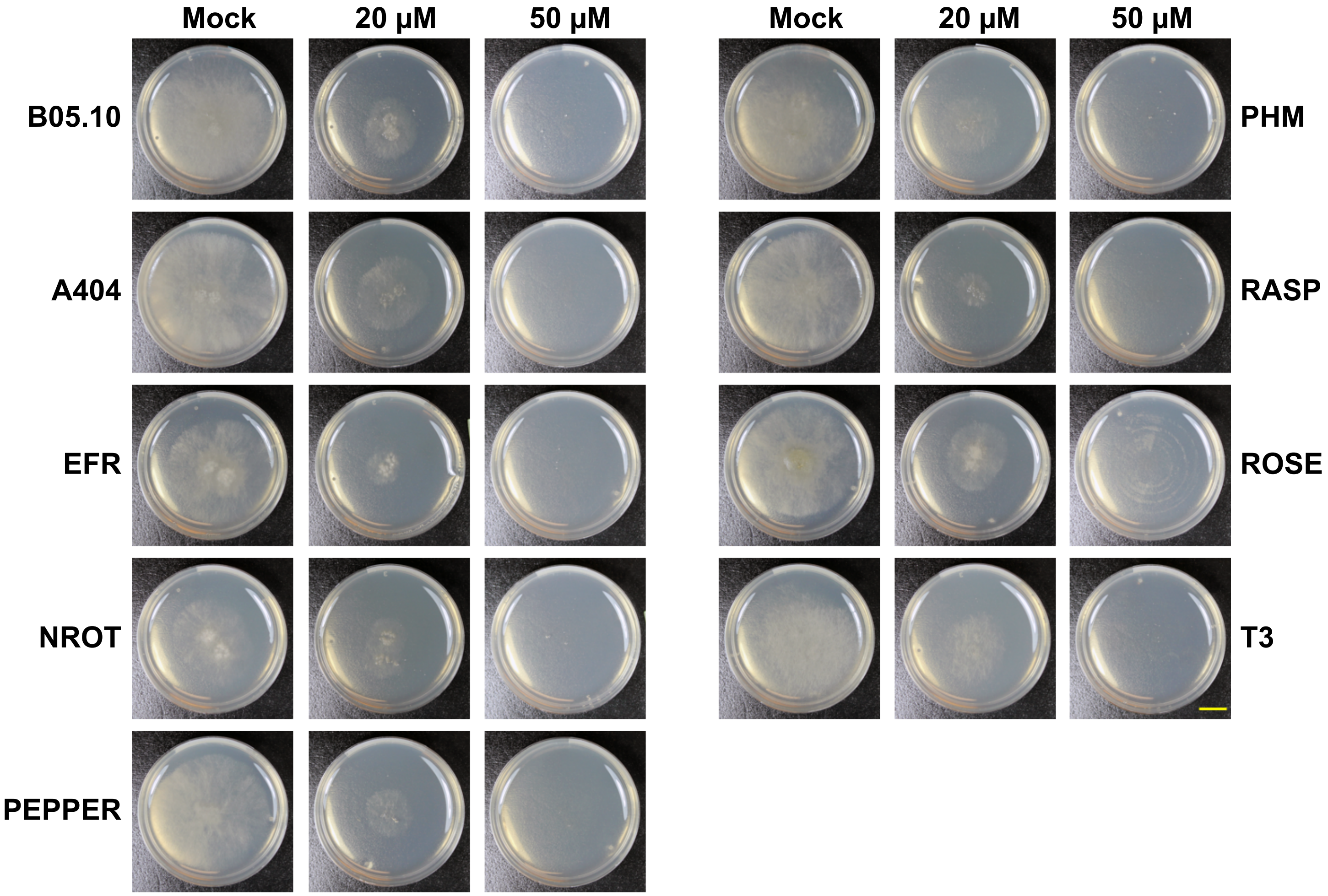

Supplementary Fig. 5. EB suppresses the germination of nine isolates of *B. cinerea*.
EB inhibits growth of various isolates of *B. cinerea*. All isolates cannot germinate under 50 µM EB. These results are consistent across two experimental replicates. Scale bar, 1 cm.

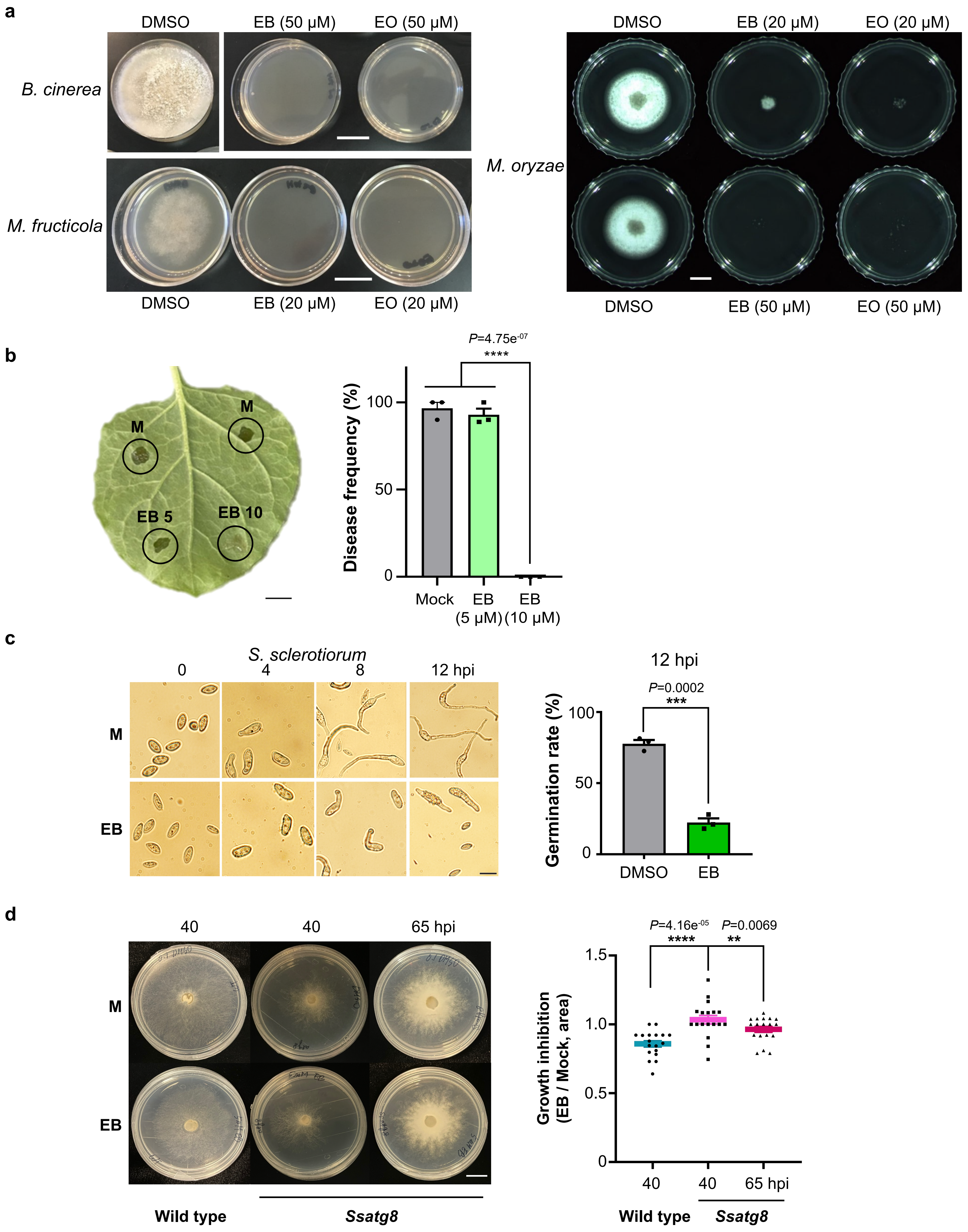
a
DMSO
EB (50 μM)
EO (50 μM)
B. cinerea
M. fructicola
DMSO
EB (20 μM)
EO (20 μM)
DMSO
EB (20 μM)
EO (20 μM)
M. oryzae
DMSO
EB (50 μM)
EO (50 μM)
b
M
M
EB 5
EB 10
P=4.75e-07
****
Disease frequency (%)
100
50
0
Mock
EB (5 μM)
EB (10 μM)
c
S. sclerotiorum
0
4
8
12 hpi
M
EB
12 hpi
P=0.0002
***
Germination rate (%)
100
50
0
DMSO
EB
d
40
40
65 hpi
M
EB
Wild type
Ssatg8
P=4.16e-05
****
P=0.0069
**
Growth inhibition (EB / Mock, area)
1.5
1.0
0.5
0
40
40
65 hpi
Wild type
Ssatg8

Supplementary Fig. 6. The effects of autophagy inhibitors EB and EO against fungal pathogens. **a,** *M. fructicola* (left bottom) is more sensitive to EB than *B. cinerea* (left top). The germination of *M. fructicola* conidia is strongly inhibited in 20 μM EB. The germination of *M. oryzae* conidia also shows severe inhibition under 50 μM EB and EO treatments (right panel). Scale bars, 1cm. **b,** Suppression of the infection of *B. cinerea* under a low-dose treatment of EB. 10 μM EB is sufficient to inhibit the infection of *B. cinerea* in *N. benthamiana* leaves. Sample sizes for analysis of the disease frequency are 86 and 43 infection spots of mock and EB treatments (either 5 or 10 μM), respectively. Graph shows mean with SE. ****, $p < 0.0001$, one-way ANOVA with Dunnett's comparison. **c,** The *S. sclerotiorum* germination is significantly inhibited under 50 μM EB at 12 hpi, compared to the mock control (M). The quantification is on the right. Graph shows mean with SE. ***, $p < 0.001$, two-tailed t-test. Scale bar, 20 μm. **d,** The hyphae growth of wild type of *S. sclerotiorum* is significantly inhibited, compared to the vegetative growth of *Ssatg8* observed under 5 μM EB. Due to difference of hyphae growth between wild type and the *Ssatg8* mutant, the growth rates of *Ssatg8* are measured at two time points (40 and 65 hpi). Graph shows means with SE. ****, $p < 0.0001$ and **, $p < 0.01$, two-way ANOVA with Dunnett's multiple comparison. Scale bar, 1 cm. The three biologically independent experiments were conducted with similar results in **a**-**d**. A representative image is shown in each panel.

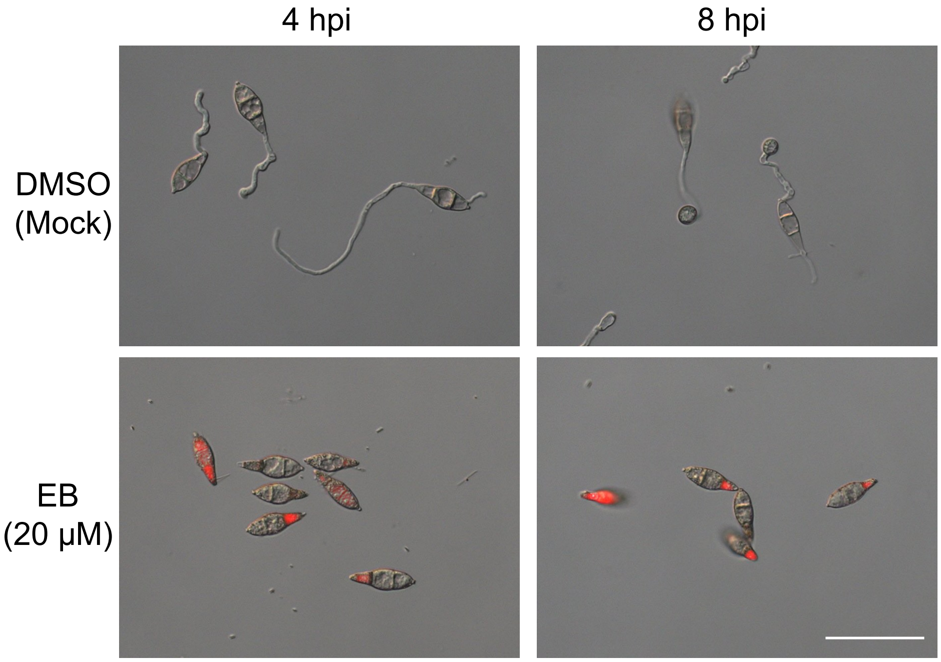

Supplementary Fig. 7. Loss of viability of *M. oryzae* conidia under EB treatment.
The *M. oryzae* conidia are dead following exposure to 20 μM EB for 4 hours, compared to the DMSO mock control. Dead conidia are confirmed by staining with propidium iodide. Red fluorescence indicates loss of membrane integrity of dead conidia under EB treatment. Scale bar, 40 μm. A representative image is shown. n=12 samples.

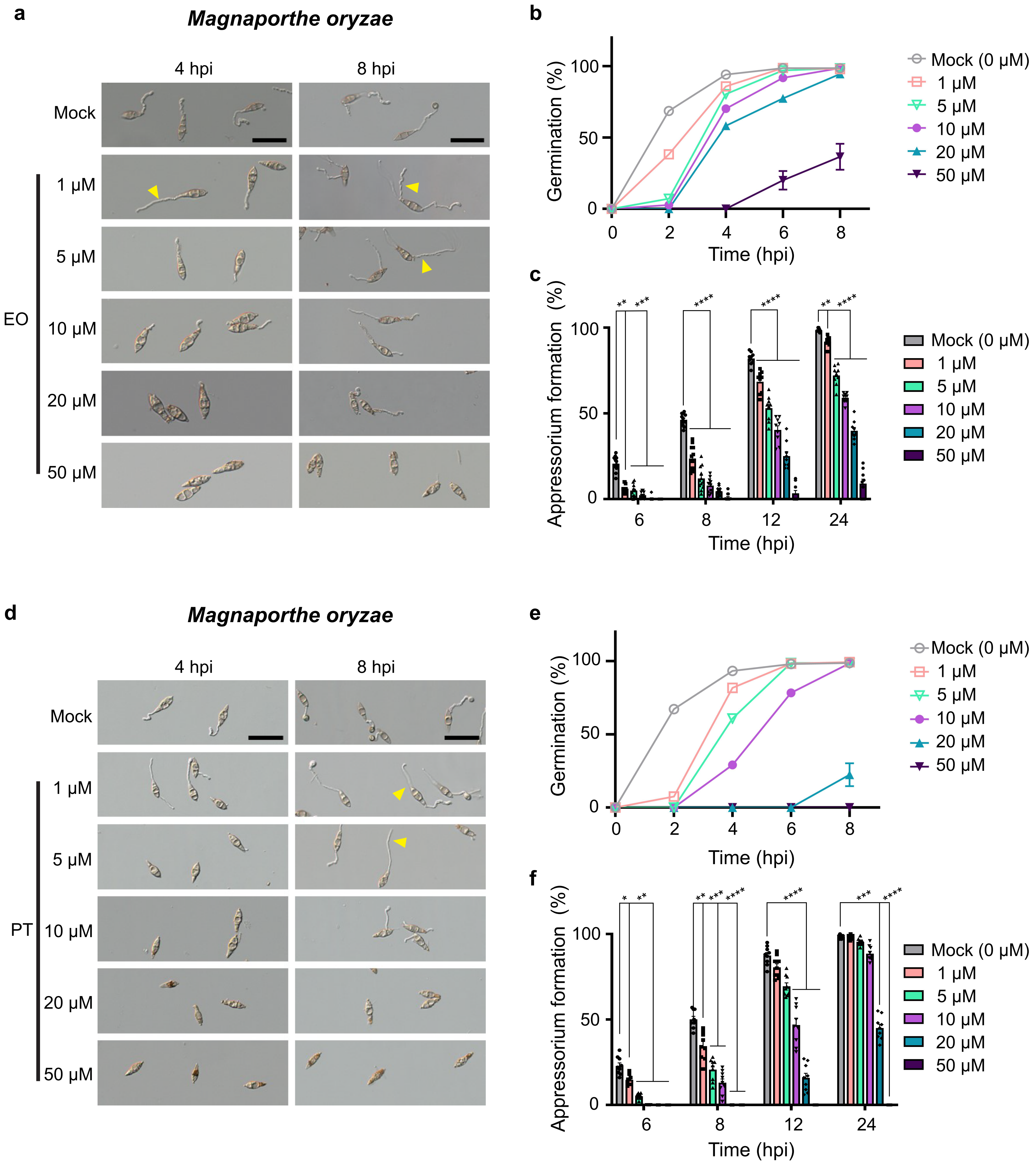

a
Magnaporthe oryzae
4 hpi
8 hpi
Mock
1 μM
5 μM
10 μM
20 μM
50 μM
EO
b
Germination (%)
Time (hpi)
Mock (0 μM)
1 μM
5 μM
10 μM
20 μM
50 μM
c
Appressorium formation (%)
Time (hpi)
Mock (0 μM)
1 μM
5 μM
10 μM
20 μM
50 μM
d
Magnaporthe oryzae
4 hpi
8 hpi
Mock
1 μM
5 μM
10 μM
20 μM
50 μM
PT
e
Germination (%)
Time (hpi)
Mock (0 μM)
1 μM
5 μM
10 μM
20 μM
50 μM
f
Appressorium formation (%)
Time (hpi)
Mock (0 μM)
1 μM
5 μM
10 μM
20 μM
50 μM

Supplementary Fig. 8. EO and PT inhibit conidial germination and appressorium formation of *M. oryzae.*

a, Suppression of conidial germination and appressorium formation of *M. oryzae* under EO treatment. **b,** The delay in germination is observed over 5 µM EO. Germination is significantly suppressed at 50 µM EO. n=3 biologically independent experiments. **c,** Appressorium formation is also inhibited at EO concentrations greater than 5 µM in a dose-dependent manner. Only 10% of conidia forms appressoria under 50 µM EO at 24 hpi. Two-way ANOVA test with Dunnett's multiple comparison. A different number of asterisks indicates statistically significant differences. n=3. **d,** Inhibition of conidial germination and appressorium formation of *M. oryzae* under PT treatment. **e,** 50 µM PT completely suppresses germination. n=3 biologically independent experiments. **f,** 50 µM PT completely inhibits appressorium formation. Two-way ANOVA test with Dunnett's multiple comparison. A different number of asterisks indicates statistically significant differences. n=3. In **a** and **d**, arrowheads indicate abnormal germ tubes of germinating conidia. Scale bars, 50 µm. All graphs show means with SE. The three biologically independent experiments were conducted with similar results in **a** and **d**. A representative image is shown in each panel. The statistical analyses are summarized in Supplementary Table 2 for **b** and **e** and Supplementary Table 3 for **c** and **f**.

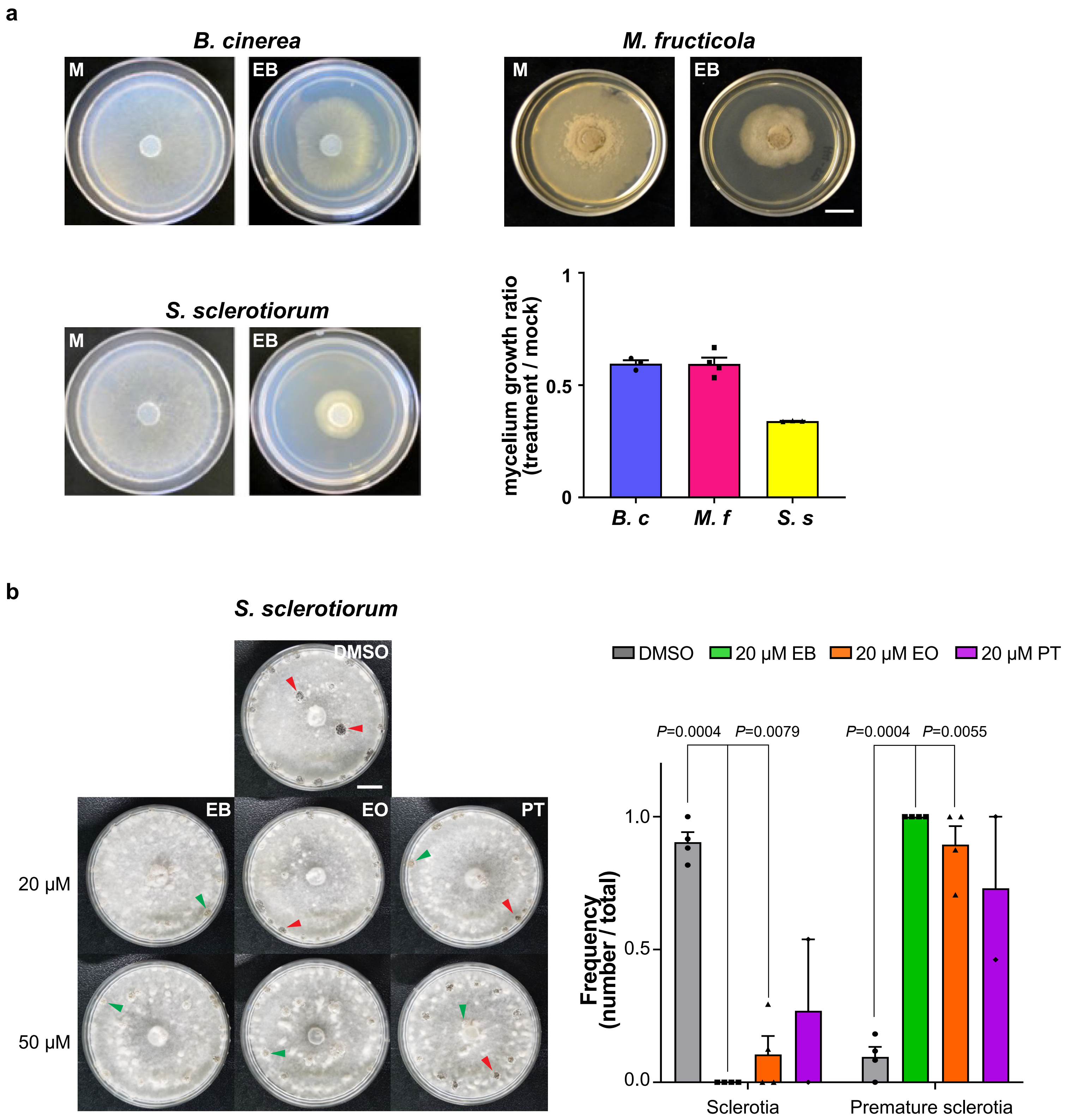
a
B. cinerea
M
EB
M. fructicola
M
EB
S. sclerotiorum
M
EB
mycelium growth ratio
(treatment / mock)
1
0.5
0
B. c
M. f
S. s
b
S. sclerotiorum
DMSO
EB
EO
PT
20 μM
50 μM
DMSO
20 μM EB
20 μM EO
20 μM PT
P=0.0004
P=0.0079
P=0.0004
P=0.0055
Frequency
(number / total)
1.0
0.5
0.0
Sclerotia
Premature sclerotia

Supplementary Fig. 9. The inhibition of mycelial growth and sclerotial development of *Ascomycota* pathogens by EB.

a, Growth retardation of mycelia of *B. cinerea*, *M. fructicola*, and *S. sclerotiorum* is observed under 50 μM EB, compared to the mock control (M). Scale bar, 2 cm. The three biologically independent experiments were conducted with similar results. A representative image is shown. **b,** *S. sclerotiorum* shows less sclerotial formation in the presence of autophagy inhibitors, EB and EO while premature sclerotia are increased. The quantification of sclerotia is shown on the right. Red and green arrowheads represent full-grown and premature sclerotia, respectively. All graphs show means with SE. Dunnett's multiple comparisons test with one-way ANOVA. n=4 for DMSO, EB, and EO and n=2 for PT. Scale bar, 2 cm.

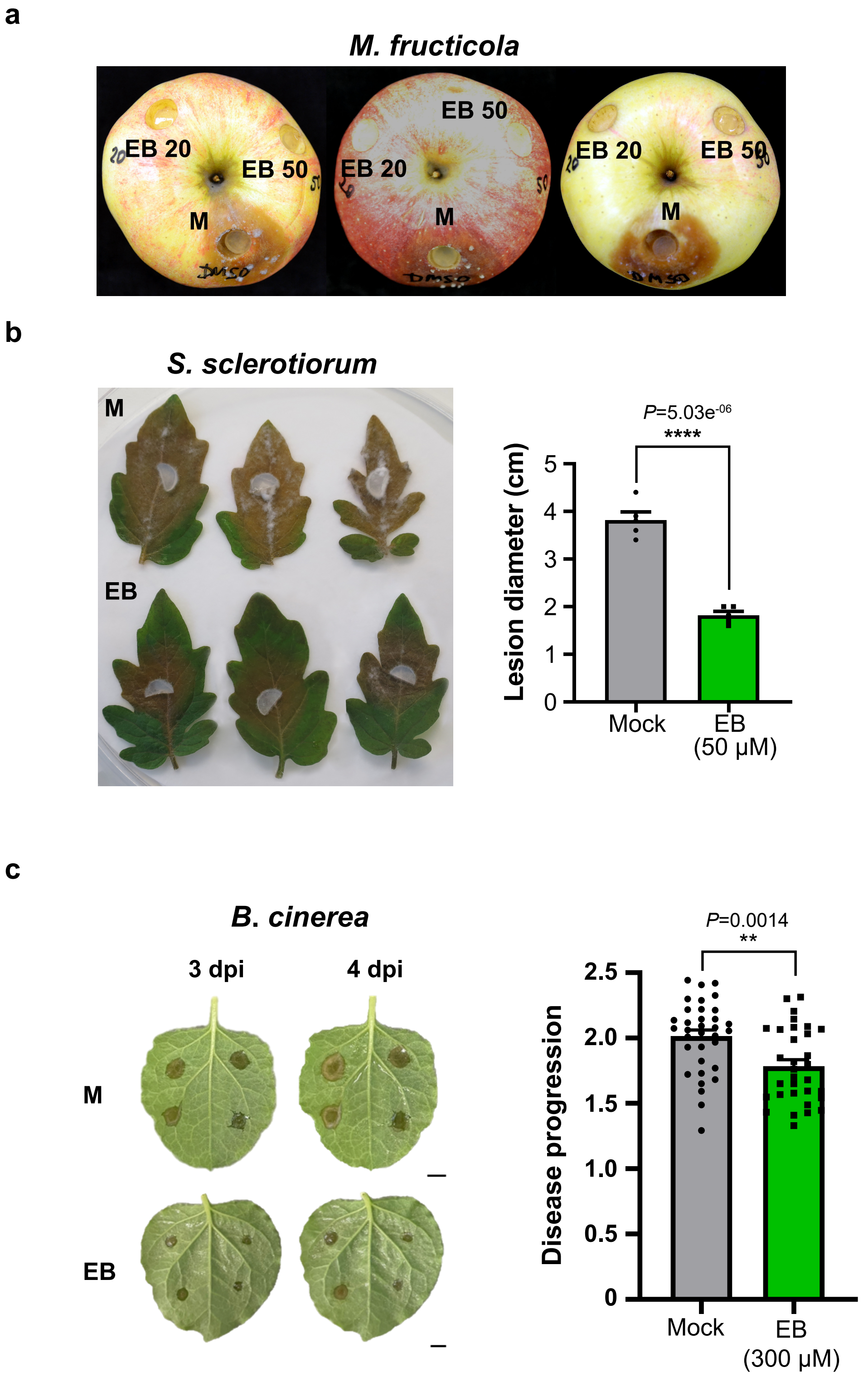

Supplementary Fig. 10. The suppression of the growth of *B. cinerea*, *M. fructicola*, and *S. sclerotiorum* on hosts by EB.

a, Growth of *M. fructicola* in the presence of two different concentrations of EB. 20 μM EB effectively inhibits the growth. **b,** Lesion diameters are significantly reduced by treatment with 50 μM EB, indicating that EB inhibits pathogenicity of *S. sclerotiorum*. Graph shows means with SE. ****, $p < 0.0001$, two-tailed t-test. **c,** The curative activity of EB against *B. cinerea*. Application of EB on the infected *N. benthamiana* leaves inhibits the disease progression. The disease progression is defined as the ratio of area of 4 dpi divided by area of 3 dpi at the same spot. Scale bars, 1 cm. Graph shows means with SE. **, $p < 0.01$, two-tailed t test. The three biologically independent experiments were conducted with similar results in **a-c**. A representative image is shown in each panel.

a

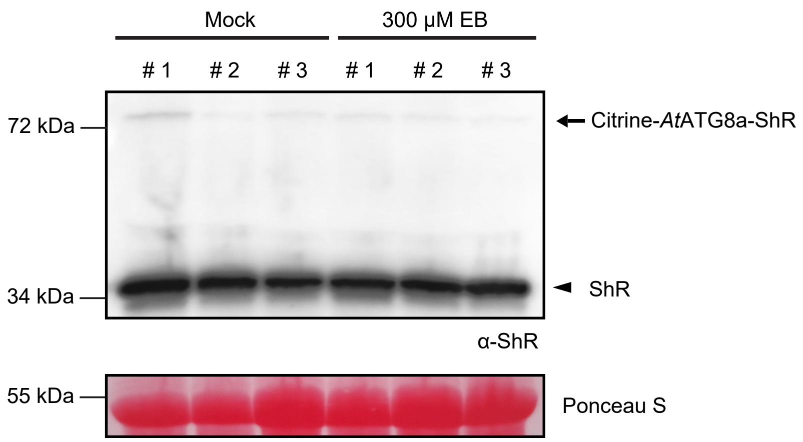

b

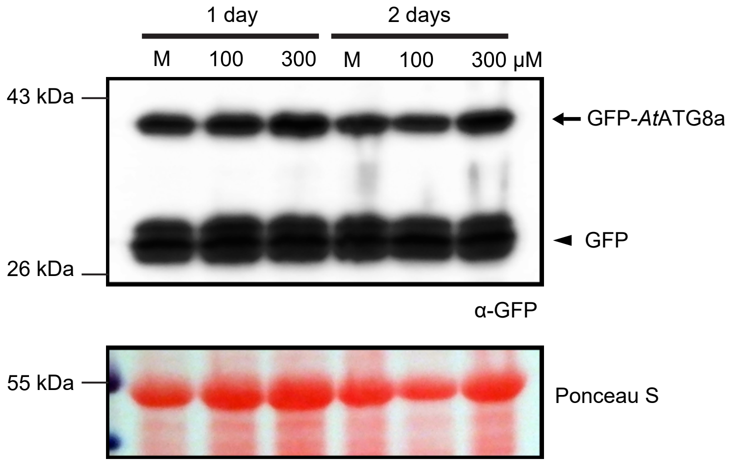

Supplementary Fig. 11. Plant ATG4-mediated processing of ATG8 is not significantly inhibited by EB treatment.

a, The effect of EB on host autophagy by multiple applications in the long-term period. 300 μM EB is applied to investigate the inhibition of host autophagy in the transgenic *N. benthamiana* expressing the *At*ATG8a-sensor once per two days for three times. 10% acetone as mock treatment and EB are applied to different leaves with a similar age in the same plants. Samples are collected for western blot at 7 dpi. Similar processing activities are observed in mock and EB treatment. An arrow indicates the unprocessed *At*ATG8-sensor while an arrowhead indicates the processed byproduct. #1, 2, and 3 indicate independent transgenic lines. **b,** Minimal effect of host autophagy under exogenous application of EB. Transgenic *Arabidopsis* expressing the *GFP-AtATG8* cassette is subjected to examine host autophagy by spray application of EB. The amount of stable GFP represents autophagy flux in plants. Host autophagy is not affected by exogenous application of EB. M indicates 1% DMSO as mock treatment. Ponceau S represents a loading control. The three biologically independent experiments were conducted with similar results in **a**-**b**. A representative image is shown in each panel.

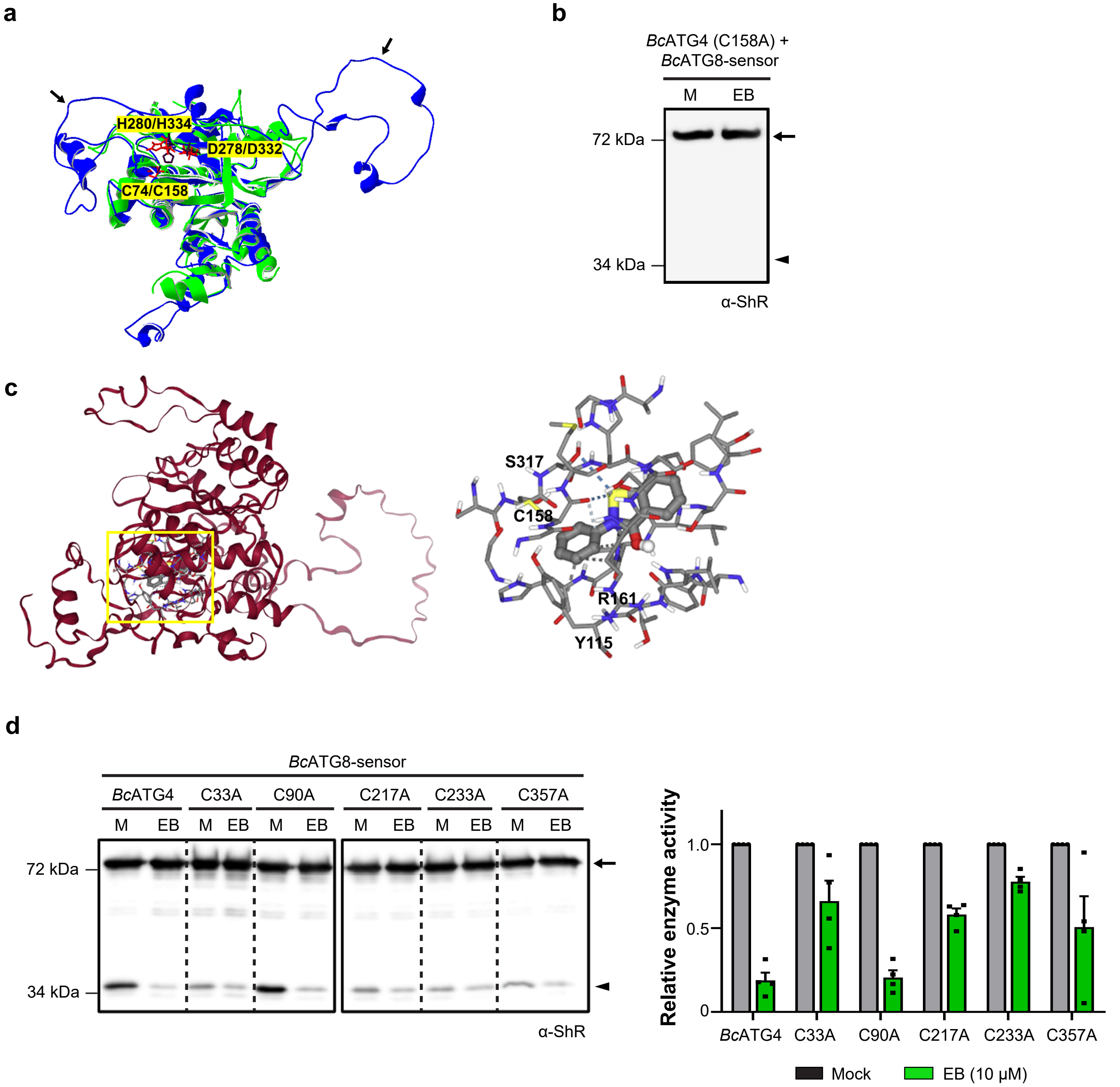

Supplementary Fig. 12. Identification of putative target cysteine residues of EB in *Bc*ATG4.
a, Superimpose between a model of *Bc*ATG4 (blue) and the crystal structure of *Hs*ATG4B (PDB, 2cy7; green). A homology model of *Bc*ATG4 was built by RoseTTAFOLD. The catalytic triads of *Hs*ATG4B and the corresponding residues in *Bc*ATG4 are labelled in yellow highlight. Arrows indicate two insertion parts in the N-terminus of *Bc*ATG4. **b,** Loss of the protease activity of the *Bc*ATG4 C158A mutant. Complete loss of the enzymatic activity of *Bc*ATG4 C158A indicates C158 is the catalytic cysteine in *Bc*ATG4. The three biologically independent experiments were conducted with similar results. A representative image is shown. A representative image is shown. **c,** Docking simulation with the model of *Bc*ATG4 and EB by AutoDock. A docking pose of EB in the model of *Bc*ATG4 (left panel) suggests EB can access the active site of *Bc*ATG4. Selected residues that interact with EB are labelled (right panel). **d,** Identification of additional putative targets of EB. The C233A mutant is the most resistant to EB, indicating a putative target of EB. The right panel is the quantification of the enzymatic activities. Arrows and arrowheads represent full-length and cleavage byproducts, respectively. The four biologically independent experiments were conducted with similar results. A representative image is shown. M and EB indicate mock and 10 μM EB treatment, respectively.

Supplementary Table 1. Statistical analyses of germination test of ***B. cinerea***

EB

**Table Analyzed**	**EB for 2way anova**
Two-way RM ANOVA	Matching: Both factors
Assume sphericity?	Yes
Alpha	0.05

Source of Variation	% of total variation	P value	P value summary	Significant?
time	30.22	<0.0001	****	Yes
concentration	35.74	<0.0001	****	Yes
time x concentration	26.12	<0.0001	****	Yes

ANOVA table	SS	DF	MS	F (DFn, DFd)	P value
time	91642	4	22911	F (4, 32) = 184.0	P=1.34e^{-21}
concentration	108392	5	21678	F (5, 40) = 101.8	P=1.17e^{-21}
time x concentration	79216	20	3961	F (20, 160) = 56.40	P=6.72e^{-62}

Data summary

Number of columns (concentration)	6
Number of rows (time)	5
Number of subjects (germinatoin %)	9
Number of missing values	0

Dunnett's multiple comparisons test
Within each row, compare columns (simple effects within rows)

Number of families	5
Number of comparisons per family	5
Alpha	0.05

	Mean Diff.	95.00% CI of diff.	Significant?	Summary	Adjusted P Value
0 time point					
0 vs. 5	0	-10.03 to 10.03	No	ns	>0.9999
0 vs. 10	0	-10.03 to 10.03	No	ns	>0.9999
0 vs. 15	0	-10.03 to 10.03	No	ns	>0.9999
0 vs. 20	0	-10.03 to 10.03	No	ns	>0.9999
0 vs. 50	0	-10.03 to 10.03	No	ns	>0.9999

2 hpi					

0 vs. 5	9.222	-0.8033 to 19.25	No	ns	0.0825
0 vs. 10	10.33	0.3078 to 20.36	Yes	*	0.0408
0 vs. 15	10.33	0.3078 to 20.36	Yes	*	0.0408
0 vs. 20	10.33	0.3078 to 20.36	Yes	*	0.0408
0 vs. 50	10.33	0.3078 to 20.36	Yes	*	0.0408

4 hpi					
0 vs. 5	41.33	31.31 to 51.36	Yes	****	<0.0001
0 vs. 10	61.89	51.86 to 71.91	Yes	****	<0.0001
0 vs. 15	63.89	53.86 to 73.91	Yes	****	<0.0001
0 vs. 20	63.89	53.86 to 73.91	Yes	****	<0.0001
0 vs. 50	63.89	53.86 to 73.91	Yes	****	<0.0001

6 hpi					
0 vs. 5	13.22	3.197 to 23.25	Yes	**	0.0047
0 vs. 10	68.89	58.86 to 78.91	Yes	****	<0.0001
0 vs. 15	80.33	70.31 to 90.36	Yes	****	<0.0001
0 vs. 20	92.22	82.20 to 102.2	Yes	****	<0.0001
0 vs. 50	95.67	85.64 to 105.7	Yes	****	<0.0001

8 hpi					
0 vs. 5	8.111	-1.914 to 18.14	No	ns	0.1549
0 vs. 10	54.11	44.09 to 64.14	Yes	****	<0.0001
0 vs. 15	75.67	65.64 to 85.69	Yes	****	<0.0001
0 vs. 20	89.89	79.86 to 99.91	Yes	****	<0.0001
0 vs. 50	100	89.97 to 110.0	Yes	****	<0.0001

EO

**Table Analyzed**	**EO for 2way anova**
Two-way RM ANOVA	Matching: Both factors
Assume sphericity?	Yes
Alpha	0.05

Source of Variation	% of total variation	P value	P value summary	Significant?
time	37.99	<0.0001	****	Yes
concentration	32.26	<0.0001	****	Yes
time x concentration	25.51	<0.0001	****	Yes

ANOVA table	SS	DF	MS	F (DFn, DFd)	P value
time	142154	4	35539	F (4, 32) = 539.6	P=7.21e^{-29}
concentration	120690	5	24138	F (5, 40) = 356.3	P=4.85e^{-32}
time x concentration	95465	20	4773	F (20, 160) = 97.75	P=6.37e^{-79}

Data summary

Number of columns (concentration)	6
Number of rows (time)	5
Number of subjects (germinatoin %)	9
Number of missing values	0

Dunnett's multiple comparisons test
Within each row, compare columns (simple effects within rows)

Number of families	5
Number of comparisons per family	5
Alpha	0.05

	Mean Diff.	95.00% CI of diff.	Significant?	Summary	Adjusted P Value
0 time point					
0 vs. 5	0	-8.360 to 8.360	No	ns	>0.9999
0 vs. 10	0	-8.360 to 8.360	No	ns	>0.9999
0 vs. 15	0	-8.360 to 8.360	No	ns	>0.9999
0 vs. 20	0	-8.360 to 8.360	No	ns	>0.9999
0 vs. 50	0	-8.360 to 8.360	No	ns	>0.9999

2 hpi					
0 vs. 5	9.444	1.085 to 17.80	Yes	*	0.0205
0 vs. 10	10.78	2.418 to 19.14	Yes	**	0.006
0 vs. 15	11	2.640 to 19.36	Yes	**	0.0048
0 vs. 20	11.11	2.751 to 19.47	Yes	**	0.0043
0 vs. 50	11.11	2.751 to 19.47	Yes	**	0.0043

4 hpi					
0 vs. 5	47.78	39.42 to 56.14	Yes	****	<0.0001
0 vs. 10	72.22	63.86 to 80.58	Yes	****	<0.0001
0 vs. 15	81.44	73.08 to 89.80	Yes	****	<0.0001
0 vs. 20	82.22	73.86 to 90.58	Yes	****	<0.0001
0 vs. 50	82.22	73.86 to 90.58	Yes	****	<0.0001

6 hpi					
0 vs. 5	16.78	8.418 to 25.14	Yes	****	<0.0001
0 vs. 10	47.67	39.31 to 56.03	Yes	****	<0.0001
0 vs. 15	81.33	72.97 to 89.69	Yes	****	<0.0001
0 vs. 20	96.22	87.86 to 104.6	Yes	****	<0.0001
0 vs. 50	100	91.64 to 108.4	Yes	****	<0.0001

8 hpi					
0 vs. 5	3.667	-4.693 to 12.03	No	ns	0.6959
0 vs. 10	13.56	5.196 to 21.92	Yes	***	0.0003
0 vs. 15	40	31.64 to 48.36	Yes	****	<0.0001
0 vs. 20	89.33	80.97 to 97.69	Yes	****	<0.0001
0 vs. 50	100	91.64 to 108.4	Yes	****	<0.0001

PT

**Table Analyzed**	**PT for 2way anova**
Two-way RM ANOVA	Matching: Both factors
Assume sphericity?	Yes
Alpha	0.05

Source of Variation	% of total variation	P value	P value summary	Significant?
time	36.81	<0.0001	****	Yes
concentration	30.53	<0.0001	****	Yes
time x concentration	26.9	<0.0001	****	Yes

ANOVA table	SS	DF	MS	F (DFn, DFd)	P value
time	130339	4	32585	F (4, 32) = 253.1	P=9.96e^{-24}
concentration	108091	5	21618	F (5, 40) = 375.9	P=1.70e^{-32}
time x concentration	95250	20	4763	F (20, 160) = 83.89	P=4.34e^{-74}

Data summary

Number of columns (concentration)	6
Number of rows (time)	5
Number of subjects (germinatoin %)	9
Number of missing values	0

Dunnett's multiple comparisons test
Within each row, compare columns (simple effects within rows)

Number of families	5
Number of comparisons per family	5
Alpha	0.05

	Mean Diff.	95.00% CI of diff.	Significant?	Summary	Adjusted P Value
0 time point					
0 vs. 5	0	-10.25 to 10.25	No	ns	>0.9999
0 vs. 10	7.105E-15	-10.25 to 10.25	No	ns	>0.9999
0 vs. 15	0	-10.25 to 10.25	No	ns	>0.9999
0 vs. 20	0	-10.25 to 10.25	No	ns	>0.9999
0 vs. 50	0	-10.25 to 10.25	No	ns	>0.9999

2 hpi					
0 vs. 5	10.22	-0.02320 to 20.47	No	ns	0.0509
0 vs. 10	10.56	0.3101 to 20.80	Yes	*	0.0393
0 vs. 15	10.56	0.3101 to 20.80	Yes	*	0.0393

0 vs. 20	10.56	0.3101 to 20.80	Yes	*	0.0393
0 vs. 50	10.56	0.3101 to 20.80	Yes	*	0.0393

4 hpi					
0 vs. 5	71.56	61.31 to 81.80	Yes	****	<0.0001
0 vs. 10	83.78	73.53 to 94.02	Yes	****	<0.0001
0 vs. 15	84.56	74.31 to 94.80	Yes	****	<0.0001
0 vs. 20	84.67	74.42 to 94.91	Yes	****	<0.0001
0 vs. 50	84.67	74.42 to 94.91	Yes	****	<0.0001

6 hpi					
0 vs. 5	30.22	19.98 to 40.47	Yes	****	<0.0001
0 vs. 10	65.22	54.98 to 75.47	Yes	****	<0.0001
0 vs. 15	91.56	81.31 to 101.8	Yes	****	<0.0001
0 vs. 20	95.22	84.98 to 105.5	Yes	****	<0.0001
0 vs. 50	98.33	88.09 to 108.6	Yes	****	<0.0001

8 hpi					
0 vs. 5	8.667	-1.579 to 18.91	No	ns	0.1489
0 vs. 10	13.89	3.643 to 24.13	Yes	**	0.0019
0 vs. 15	45	34.75 to 55.25	Yes	****	<0.0001
0 vs. 20	82	71.75 to 92.25	Yes	****	<0.0001
0 vs. 50	100	89.75 to 110.2	Yes	****	<0.0001

Supplementary Table 2. Statistical analyses of germination test of ***M. oryzae***

EB

**Table Analyzed**	**EB for 2way anova**
Two-way RM ANOVA	Matching: Both factors
Assume sphericity?	Yes
Alpha	0.05

Source of Variation	% of total variation	P value	P value summary	Significant?
time	30.2	<0.0001	****	Yes
concentration	39.49	<0.0001	****	Yes
time x concentration	19.91	<0.0001	****	Yes

ANOVA table	SS	DF	MS	F (DFn, DFd)	P value
time	251278	4	62819	F (4, 44) = 536.1	P=1.06e^{-36}
concentration	328635	6	54772	F (6, 66) = 131.1	P=1.08e^{-34}
time x concentration	165659	24	6902	F (24, 264) = 39.18	P=5.72e^{-73}

Data summary

Number of columns (concentration)	7
Number of rows (time)	5
Number of subjects (germinatoin %)	12
Number of missing values	0

Dunnett's multiple comparisons test
Within each row, compare columns (simple effects within rows)

Number of families	5
Number of comparisons per family	6
Alpha	0.05

	Mean Diff.	95.00% CI of diff.	Significant?	Summary	Adjusted P Value
0 time point					
1% DMSO vs. 1µM	0	-14.00 to 14.00	No	ns	>0.9999
1% DMSO vs. 3µM	0	-14.00 to 14.00	No	ns	>0.9999
1% DMSO vs. 5µM	0	-14.00 to 14.00	No	ns	>0.9999
1% DMSO vs. 7µM	0	-14.00 to 14.00	No	ns	>0.9999
1% DMSO vs. 10µM	0	-14.00 to 14.00	No	ns	>0.9999
1% DMSO vs. 20µM	0	-14.00 to 14.00	No	ns	>0.9999

2 hpi					
1% DMSO vs. 1µM	0	-14.00 to 14.00	No	ns	>0.9999
1% DMSO vs. 3µM	0	-14.00 to 14.00	No	ns	>0.9999
1% DMSO vs. 5µM	0	-14.00 to 14.00	No	ns	>0.9999
1% DMSO vs. 7µM	0	-14.00 to 14.00	No	ns	>0.9999
1% DMSO vs. 10µM	0	-14.00 to 14.00	No	ns	>0.9999
1% DMSO vs. 20µM	0	-14.00 to 14.00	No	ns	>0.9999

4 hpi					
1% DMSO vs. 1µM	6.333	-7.666 to 20.33	No	ns	0.7072
1% DMSO vs. 3µM	14.25	0.2504 to 28.25	Yes	*	0.0443
1% DMSO vs. 5µM	64.92	50.92 to 78.92	Yes	****	<0.0001
1% DMSO vs. 7µM	87.17	73.17 to 101.2	Yes	****	<0.0001
1% DMSO vs. 10µM	94.33	80.33 to 108.3	Yes	****	<0.0001
1% DMSO vs. 20µM	94.33	80.33 to 108.3	Yes	****	<0.0001

6 hpi					
1% DMSO vs. 1µM	4	-10.00 to 18.00	No	ns	0.9461
1% DMSO vs. 3µM	5.583	-8.416 to 19.58	No	ns	0.8023
1% DMSO vs. 5µM	29.25	15.25 to 43.25	Yes	****	<0.0001
1% DMSO vs. 7µM	57.33	43.33 to 71.33	Yes	****	<0.0001
1% DMSO vs. 10µM	99.67	85.67 to 113.7	Yes	****	<0.0001
1% DMSO vs. 20µM	100	86.00 to 114.0	Yes	****	<0.0001

8 hpi					
1% DMSO vs. 1µM	0	-14.00 to 14.00	No	ns	>0.9999
1% DMSO vs. 3µM	0.5833	-13.42 to 14.58	No	ns	0.9999
1% DMSO vs. 5µM	2.5	-11.50 to 16.50	No	ns	0.9944
1% DMSO vs. 7µM	49.92	35.92 to 63.92	Yes	****	<0.0001
1% DMSO vs. 10µM	89.75	75.75 to 103.7	Yes	****	<0.0001
1% DMSO vs. 20µM	100	86.00 to 114.0	Yes	****	<0.0001

EO

**Table Analyzed**	**EO for 2way anova**
Two-way RM ANOVA	Matching: Both factors
Assume sphericity?	Yes
Alpha	0.05

Source of Variation	% of total variation	P value	P value summary	Significant?
time	66.14	<0.0001	****	Yes
concentration	19.63	<0.0001	****	Yes
time x concentration	10.56	<0.0001	****	Yes

ANOVA table	SS	DF	MS	F (DFn, DFd)	P value
time	324838	4	81210	F (4, 32) = 1240	P=1.37e^{-34}
concentration	96411	5	19282	F (5, 40) = 288.1	P=3.04e^{-30}
time x concentration	51839	20	2592	F (20, 160) = 44.32	P=8.26e^{-55}

Data summary

Number of columns (concentration)	6
Number of rows (time)	5
Number of subjects (germinatoin %)	9
Number of missing values	0

Dunnett's multiple comparisons test
Within each row, compare columns (simple effects within rows)

Number of families	5
Number of comparisons per family	5
Alpha	0.05

	Mean Diff.	95.00% CI of diff.	Significant?	Summary	Adjusted P Value
0 time point					
1% DMSO vs. 1µM	0	-9.149 to 9.149	No	ns	>0.9999
1% DMSO vs. 5µM	0	-9.149 to 9.149	No	ns	>0.9999
1% DMSO vs. 10µM	0	-9.149 to 9.149	No	ns	>0.9999
1% DMSO vs. 20µM	0	-9.149 to 9.149	No	ns	>0.9999
1% DMSO vs. 50µM	0	-9.149 to 9.149	No	ns	>0.9999

2 hpi					
1% DMSO vs. 1µM	23.67	14.52 to 32.82	Yes	****	<0.0001
1% DMSO vs. 5µM	51.33	42.18 to 60.48	Yes	****	<0.0001
1% DMSO vs. 10µM	62.22	53.07 to 71.37	Yes	****	<0.0001
1% DMSO vs. 20µM	71.56	62.41 to 80.70	Yes	****	<0.0001
1% DMSO vs. 50µM	71.56	62.41 to 80.70	Yes	****	<0.0001

4 hpi					
1% DMSO vs. 1µM	8.889	-0.2599 to 18.04	No	ns	0.06
1% DMSO vs. 5µM	15.67	6.518 to 24.82	Yes	***	0.0001
1% DMSO vs. 10µM	21.67	12.52 to 30.82	Yes	****	<0.0001
1% DMSO vs. 20µM	37.22	28.07 to 46.37	Yes	****	<0.0001
1% DMSO vs. 50µM	95.44	86.30 to 104.6	Yes	****	<0.0001

6 hpi					
1% DMSO vs. 1µM	-0.1111	-9.260 to 9.038	No	ns	>0.9999
1% DMSO vs. 5µM	1.333	-7.815 to 10.48	No	ns	0.9955
1% DMSO vs. 10µM	2.889	-6.260 to 12.04	No	ns	0.8896
1% DMSO vs. 20µM	18.89	9.740 to 28.04	Yes	****	<0.0001
1% DMSO vs. 50µM	72.56	63.41 to 81.70	Yes	****	<0.0001

8 hpi					
1% DMSO vs. 1µM	-0.1111	-9.260 to 9.038	No	ns	>0.9999
1% DMSO vs. 5µM	-0.3333	-9.482 to 8.815	No	ns	0.9999
1% DMSO vs. 10µM	-0.3333	-9.482 to 8.815	No	ns	0.9999
1% DMSO vs. 20µM	5.556	-3.593 to 14.70	No	ns	0.3972
1% DMSO vs. 50µM	52	42.85 to 61.15	Yes	****	<0.0001

PT

**Table Analyzed**	**PT for 2way anova**
Two-way RM ANOVA	Matching: Both factors
Assume sphericity?	Yes
Alpha	0.05

Source of Variation	% of total variation	P value	P value summary	Significant?
time	26.41	<0.0001	****	Yes
concentration	37.46	<0.0001	****	Yes
time x concentration	18.27	<0.0001	****	Yes

ANOVA table	SS	DF	MS	F (DFn, DFd)	P value
time	127374	4	31843	F (4, 32) = 72.07	P=1.51e^{-15}
concentration	180661	5	36132	F (5, 40) = 54.10	P=9.59e^{-17}
time x concentration	88123	20	4406	F (20, 160) = 27.32	P=1.65e^{-41}

Data summary

Number of columns (concentration)	6
Number of rows (time)	5
Number of subjects (germinatoin %)	9
Number of missing values	0

Dunnett's multiple comparisons test
Within each row, compare columns (simple effects within rows)

Number of families	5
Number of comparisons per family	5
Alpha	0.05

	Mean Diff.	95.00% CI of diff.	Significant?	Summary	Adjusted P Value
0 time point					
1% DMSO vs. 1µM	0	-17.27 to 17.27	No	ns	>0.9999
1% DMSO vs. 5µM	0	-17.27 to 17.27	No	ns	>0.9999
1% DMSO vs. 10µM	0	-17.27 to 17.27	No	ns	>0.9999
1% DMSO vs. 20µM	0	-17.27 to 17.27	No	ns	>0.9999
1% DMSO vs. 50µM	0	-17.27 to 17.27	No	ns	>0.9999

2 hpi					
1% DMSO vs. 1µM	67.89	50.62 to 85.16	Yes	****	<0.0001
1% DMSO vs. 5µM	72.89	55.62 to 90.16	Yes	****	<0.0001
1% DMSO vs. 10µM	72.89	55.62 to 90.16	Yes	****	<0.0001

1% DMSO vs. 20µM	72.89	55.62 to 90.16	Yes	****	<0.0001
1% DMSO vs. 50µM	72.89	55.62 to 90.16	Yes	****	<0.0001

4 hpi					
1% DMSO vs. 1µM	32.78	15.51 to 50.05	Yes	****	<0.0001
1% DMSO vs. 5µM	50.89	33.62 to 68.16	Yes	****	<0.0001
1% DMSO vs. 10µM	73.67	56.40 to 90.94	Yes	****	<0.0001
1% DMSO vs. 20µM	93.56	76.29 to 110.8	Yes	****	<0.0001
1% DMSO vs. 50µM	93.56	76.29 to 110.8	Yes	****	<0.0001

6 hpi					
1% DMSO vs. 1µM	6.111	-11.16 to 23.38	No	ns	0.9104
1% DMSO vs. 5µM	32	14.73 to 49.27	Yes	****	<0.0001
1% DMSO vs. 10µM	44.33	27.06 to 61.60	Yes	****	<0.0001
1% DMSO vs. 20µM	98.56	81.29 to 115.8	Yes	****	<0.0001
1% DMSO vs. 50µM	98.67	81.40 to 115.9	Yes	****	<0.0001

8 hpi					
1% DMSO vs. 1µM	4.222	-13.05 to 21.49	No	ns	0.9811
1% DMSO vs. 5µM	32.56	15.29 to 49.83	Yes	****	<0.0001
1% DMSO vs. 10µM	32.44	15.17 to 49.71	Yes	****	<0.0001
1% DMSO vs. 20µM	93.44	76.17 to 110.7	Yes	****	<0.0001
1% DMSO vs. 50µM	99.22	81.95 to 116.5	Yes	****	<0.0001

Supplementary Table 3. Statistical analyses of appressorium formation test of *M. oryzae*

EB

Table Analyzed	EB for 2way anova
Because some values are missing, these data were analyzed by fitting a mixed model, rather than by repeated measures ANOVA (which can't handle missing values).<br>Note that the data table contains missing values. These mixed model results will be meaningful only if those values are missing for completely random reasons. If the reason that a value is missing is related to what value might have been, then these results are misleading.	

Mixed-effects model (REML)	Matching: Both factors
Assume sphericity?	No
Alpha	0.05

Fixed effects (type III)	P value	P value summary	Statistically significant (P < 0.05)?	F (DFn, DFd)	Geisser-Greenhouse's epsilon
Time	P=1.44e^{-16}	****	Yes	F (3.000, 33.00) = 98.82	1
Concentration	P=5.28e^{-20}	****	Yes	F (6.000, 66.00) = 39.51	1
Time x Concentration	P=1.62e^{-19}	****	Yes	F (18.00, 132.0) = 11.68	1

Random effects	SD	Variance
Subject	11.19	125.3
Subject x Time	1.405	1.975
Subject x Concentration	10.66	113.6
Residual	9.463	89.54

Was the matching effective?

Chi-square, df	187.9, 3
P value	<0.0001
P value summary	****
Is there significant matching (P < 0.05)?	Yes

Data summary

Number of columns (Concentration)	7
Number of rows (Time)	4
Number of subjects (Subject)	12
Number of missing values	66

Dunnett's multiple comparisons test

Number of families	4
Number of comparisons per family	6
Alpha	0.05

	Mean Diff.	95.00% CI of diff.	Significant?	Summary	Adjusted P Value
6 hpi					
1% DMSO vs. 1 µM	10.28	-3.114 to 23.67	No	ns	0.1463
1% DMSO vs. 3 µM	15.72	1.334 to 30.11	Yes	*	0.0327
1% DMSO vs. 5 µM	22.75	0.8962 to 44.60	Yes	*	0.0405
1% DMSO vs. 7 µM	28.5	8.584 to 48.42	Yes	**	0.0076
1% DMSO vs. 10 µM	29.5	8.949 to 50.05	Yes	**	0.0055
1% DMSO vs. 20 µM	29.5	8.949 to 50.05	Yes	**	0.0055
8 hpi					
1% DMSO vs. 1 µM	11.03	-12.77 to 34.83	No	ns	0.5266
1% DMSO vs. 3 µM	14.25	-11.63 to 40.13	No	ns	0.3775
1% DMSO vs. 5 µM	29.67	-2.776 to 62.11	No	ns	0.0772
1% DMSO vs. 7 µM	32.25	7.535 to 56.97	Yes	*	0.0129
1% DMSO vs. 10 µM	51.25	23.44 to 79.06	Yes	***	0.0008
1% DMSO vs. 20 µM	51.25	23.44 to 79.06	Yes	***	0.0008
12 hpi					
1% DMSO vs. 1 µM	27.33	-25.00 to 79.67	No	ns	0.3357
1% DMSO vs. 3 µM	36.33	8.060 to 64.61	Yes	*	0.0188
1% DMSO vs. 5 µM	44	6.894 to 81.11	Yes	*	0.026
1% DMSO vs. 7 µM	56.44	29.60 to 83.29	Yes	**	0.0022
1% DMSO vs. 10 µM	67.08	51.17 to 83.00	Yes	****	<0.0001
1% DMSO vs. 20 µM	78	59.58 to 96.42	Yes	****	<0.0001
24 hpi					
1% DMSO vs. 1 µM	38.5	-15.81 to 92.81	No	ns	0.1594
1% DMSO vs. 3 µM	55.5	18.05 to 92.95	Yes	*	0.0101
1% DMSO vs. 5 µM	62.17	15.84 to 108.5	Yes	*	0.0155
1% DMSO vs. 7 µM	74.5	41.94 to 107.1	Yes	**	0.0014
1% DMSO vs. 10 µM	82.67	56.88 to 108.5	Yes	***	0.0003
1% DMSO vs. 20 µM	100		Yes	****	<0.0001

EO

Table Analyzed	EO for 2way anova
Because some values are missing, these data were analyzed by fitting a mixed model, rather than by repeated measures ANOVA (which can't handle missing values).<br>Note that the data table contains missing values. These mixed model results will be meaningful only if those values are missing for completely random reasons. If the reason that a value is missing is related to what value might have been, then these results are misleading.	

Mixed-effects model (REML)	Matching: Both factors
Assume sphericity?	No
Alpha	0.05

Fixed effects (type III)	P value	P value summary	Statistically significant (P < 0.05)?	F (DFn, DFd)	Geisser-Greenhouse's epsilon
Time	$P=1.31e^{-09}$	****	Yes	F (1.259, 13.85) = 226.7	0.4198
Concentration	$P=1.25e^{-10}$	****	Yes	F (1.716, 18.87) = 170.9	0.3432
Time x Concentration	$P=8.14e^{-12}$	****	Yes	F (3.067, 23.92) = 71.58	0.2045

Random effects	SD	Variance
Subject	4.681	21.91
Subject x Time	4.683	21.93
Subject x Concentration	4.682	21.92
Residual	4.682	21.92

Was the matching effective?

Chi-square, df	128.4, 3
P value	<0.0001
P value summary	****
Is there significant matching (P < 0.05)?	Yes

Data summary

Number of columns (Concentration)	6
Number of rows (Time)	4
Number of subjects (Subject)	12
Number of missing values	48

Dunnett's multiple comparisons test

Number of families	4
Number of comparisons per family	5
Alpha	0.05

	Mean Diff.	95.00% CI of diff.	Significant?	Summary	Adjusted P Value
6 hpi					
1% DMSO vs. 1 µM	22.67	8.086 to 37.25	Yes	**	0.0032
1% DMSO vs. 5 µM	20.33	10.43 to 30.24	Yes	***	0.0004
1% DMSO vs. 10 µM	24	12.68 to 35.32	Yes	***	0.0003
1% DMSO vs. 20 µM	29.97	18.44 to 41.50	Yes	***	0.0002
1% DMSO vs. 50 µM	30.42	18.76 to 42.07	Yes	***	0.0002
8 hpi					
1% DMSO vs. 1 µM	24.92	14.73 to 35.10	Yes	****	<0.0001
1% DMSO vs. 5 µM	34.17	26.39 to 41.95	Yes	****	<0.0001
1% DMSO vs. 10 µM	37.58	27.06 to 48.11	Yes	****	<0.0001
1% DMSO vs. 20 µM	48.97	38.99 to 58.95	Yes	****	<0.0001
1% DMSO vs. 50 µM	53.08	43.20 to 62.97	Yes	****	<0.0001
12 hpi					
1% DMSO vs. 1 µM	13.56	9.154 to 17.96	Yes	****	<0.0001
1% DMSO vs. 5 µM	29	22.94 to 35.06	Yes	****	<0.0001
1% DMSO vs. 10 µM	41.67	36.12 to 47.21	Yes	****	<0.0001
1% DMSO vs. 20 µM	56.89	48.27 to 65.50	Yes	****	<0.0001
1% DMSO vs. 50 µM	78.67	72.45 to 84.89	Yes	****	<0.0001
24 hpi					
1% DMSO vs. 1 µM	6.556	2.824 to 10.29	Yes	**	0.0022
1% DMSO vs. 5 µM	26.33	20.54 to 32.12	Yes	****	<0.0001
1% DMSO vs. 10 µM	39.33	35.50 to 43.17	Yes	****	<0.0001
1% DMSO vs. 20 µM	58.56	52.79 to 64.32	Yes	****	<0.0001
1% DMSO vs. 50 µM	89.44	81.42 to 97.46	Yes	****	<0.0001

PT

Table Analyzed	PT for 2way anova
Because some values are missing, these data were analyzed by fitting a mixed model, rather than by repeated measures ANOVA (which can't handle missing values).<br>Note that the data table contains missing values. These mixed model results will be meaningful only if those values are missing for completely random reasons. If the reason that a value is missing is related to what value might have been, then these results are misleading.	

Mixed-effects model (REML)	Matching: Both factors
Assume sphericity?	No
Alpha	0.05

Fixed effects (type III)	P value	P value summary	Statistically significant (P < 0.05)?	F (DFn, DFd)	Geisser-Greenhouse's epsilon
Time	P=2.64e^{-05}	****	Yes	F (1.099, 8.795) = 73.49	0.3665
Concentration	P=7.31e^{-06}	****	Yes	F (1.158, 9.262) = 84.12	0.2315
Time x Concentration	P=0.00015	***	Yes	F (1.426, 9.129) = 39.03	0.0951

Random effects	SD	Variance
Subject	0	0
Subject x Time	7.9	62.41
Subject x Concentration	7.562	57.18
Residual	7.301	53.31

Was the matching effective?

Chi-square, df	58.1, 2
P value	<0.0001
P value summary	****
Is there significant matching (P < 0.05)?	Yes

Data summary

Number of columns (Concentration)	6
Number of rows (Time)	4
Number of subjects (Subject)	9
Number of missing values	24

Dunnett's multiple comparisons test

Number of families	4
Number of comparisons per family	5
Alpha	0.05

	Mean Diff.	95.00% CI of diff.	Significant?	Summary	Adjusted P Value
6 hpi					
1% DMSO vs. 1 µM	16.28	1.320 to 31.24	Yes	*	0.0364
1% DMSO vs. 5 µM	26	8.013 to 43.99	Yes	**	0.0074
1% DMSO vs. 10 µM	29.44	13.92 to 44.97	Yes	**	0.0014
1% DMSO vs. 20 µM	29.44	13.92 to 44.97	Yes	**	0.0014
1% DMSO vs. 50 µM	29.44	13.92 to 44.97	Yes	**	0.0014
8 hpi					
1% DMSO vs. 1 µM	24.72	13.88 to 35.57	Yes	**	0.0015
1% DMSO vs. 5 µM	39.67	21.77 to 57.56	Yes	***	0.0005
1% DMSO vs. 10 µM	46.33	27.24 to 65.42	Yes	***	0.0003
1% DMSO vs. 20 µM	54.89	42.47 to 67.31	Yes	****	<0.0001
1% DMSO vs. 50 µM	54.89	42.47 to 67.31	Yes	****	<0.0001
12 hpi					
1% DMSO vs. 1 µM	4.667	-9.208 to 18.54	No	ns	0.65
1% DMSO vs. 5 µM	17.17	-0.5093 to 34.84	No	ns	0.0556
1% DMSO vs. 10 µM	47.83	42.71 to 52.96	Yes	****	<0.0001
1% DMSO vs. 20 µM	77.17	70.00 to 84.33	Yes	****	<0.0001
1% DMSO vs. 50 µM	88.33	79.86 to 96.81	Yes	****	<0.0001
24 hpi					
1% DMSO vs. 1 µM	0.4444	-2.332 to 3.221	No	ns	0.9603
1% DMSO vs. 5 µM	18	-7.908 to 43.91	No	ns	0.1978
1% DMSO vs. 10 µM	35.11	-6.708 to 76.93	No	ns	0.1035
1% DMSO vs. 20 µM	68.67	42.98 to 94.35	Yes	***	0.0001
1% DMSO vs. 50 µM	98.78	97.52 to 100.0	Yes	****	<0.0001
